# Shotgun metagenomics reveals interkingdom association between intestinal bacteria and fungi involving competition for nutrients

**DOI:** 10.1186/s40168-023-01693-w

**Published:** 2023-12-14

**Authors:** Zixuan Xie, Aleix Canalda-Baltrons, Christophe d’Enfert, Chaysavanh Manichanh

**Affiliations:** 1 Microbiome Lab, Vall d’Hebron Institut de Recerca (VHIR), Vall d’Hebron Barcelona Hospital Campus, Barcelona, Spain; 2 Departament de Medicina, Universitat Autònoma de Barcelona, Barcelona, Spain; 3 Institut Pasteur, Université Paris Cité, INRAE USC2019, Unité Biologie et Pathogénicité Fongiques, Paris, France

**Keywords:** Mycobiome, Microbiome, Diet, Shotgun metagenomes, Comprehensive database

## Abstract

**Background:**

The accuracy of internal-transcribed-spacer (ITS) and shotgun metagenomics has not been robustly evaluated, and the effect of diet on the composition and function of the bacterial and fungal gut microbiome in a longitudinal setting has been poorly investigated. Here we compared two approaches to study the fungal community (ITS and shotgun metagenomics), proposed an enrichment protocol to perform a reliable mycobiome analysis using a comprehensive in-house fungal database, and correlated dietary data with both bacterial and fungal communities.

**Results:**

We found that shotgun DNA sequencing after a new enrichment protocol combined with the most comprehensive and novel fungal databases provided a cost-effective approach to perform gut mycobiome profiling at the species level and to integrate bacterial and fungal community analyses in fecal samples. The mycobiome was significantly more variable than the bacterial community at the compositional and functional levels. Notably, we showed that microbial diversity, composition, and functions were associated with habitual diet composition instead of driven by global dietary changes. Our study indicates a potential competitive inter-kingdom interaction between bacteria and fungi for food foraging.

**Conclusion:**

Together, our present work proposes an efficient workflow to study the human gut microbiome integrating robustly fungal, bacterial, and dietary data. These findings will further advance our knowledge of the interaction between gut bacteria and fungi and pave the way for future investigations in human mycobiome.

Video Abstract

**Supplementary Information:**

The online version contains supplementary material available at 10.1186/s40168-023-01693-w.

## Introduction

The fungal microbiome, named mycobiome, is believed to play essential roles in human health and disease [1–10]. However, in comparison with the gut bacterial microbiome, the human gut mycobiome has only been partially investigated (225 results of “human gut mycobiome”, 32,653 results of “human gut microbiome” in PubMed before February 2023). The knowledge gap could reflect the lack of a comprehensive fungal sequence database and bioinformatics pipeline. However, we have recently addressed this issue by creating a gene catalog with over 1.6 million fungal single-copy marker genes and 3 million fungal protein sequences. Additionally, we developed a tool that enables fungal taxonomic and functional profiling from shotgun metagenomic sequence data [11]. Another reason for this knowledge gap may lie in the sequencing approach that has been so far used to access the composition of the fungal microbiome. Indeed, similar to the analysis of the human gut bacterial microbiome, studies of the human gut mycobiome have frequently exploited amplicon sequencing of ribosomal DNA, especially the sequencing of the internal transcribed spacer (ITS) region [6, 8, 10, 12, 13]. However, it has been demonstrated that the copy numbers of the ribosomal DNA in fungi can vary widely at the species level and even at the strain level [14–18]. This high variation challenges the quantitative taxonomic profiling of fungal communities of an environment. Moreover, such as other amplicon sequencing approaches, ITS, or 18S sequencing methods have been criticized for having a low phylogenetic resolution at the species level [12, 15, 19], and for the disability to provide functional information [20]. To address the above issues, combining shotgun metagenomic sequencing which randomly breaks and sequences the whole genomic DNA with the use of single-copy marker genes [11] could be a more suitable strategy.

The microbial species in the human gut display varying abundance levels, of which the fungal species only make up a small proportion. The cultivable fungi in feces range from 10^2^ to 10^7^ cfu/g [21–23], indicating that the ratio of the fungal cells against the bacterial cells is between 10^−9^ and 10^−4^ [24]. Moreover, the proportion of the fungal genes in the human gut is reported to be less than 0.08% of the whole microbial metagenome [11, 25, 26]. This numerical inferiority, making the human gut mycobiome a subdominant community, is a potential source of bias when using shotgun sequencing to recover fungal sequences, as this approach provides compositional data. Thus, either deep shotgun sequencing, which has a relatively high cost, or an experimental fungal enrichment protocol can provide a solution to this issue. It is known that the size of fungal cells is generally larger than that of bacterial cells. The diameter of fungal yeasts is approximately 2–10 μm, and the diameter of hyphae can reach 40 μm [27–30], while the diameter of bacterial cells is normally between 0.2 and 2 µm [31]. Thus, centrifugation that separates cells from these two kingdoms based on differentiated cell sizes could be an option to enrich the fungal cells in human fecal samples.

Therefore, in this study, we have introduced an enrichment protocol that separated the original human fecal samples into two separate partitions and successfully enriched fungal and bacterial cells respectively. This protocol has been validated on a cohort of 48 fecal samples collected from 6 healthy volunteers longitudinally within 8 weeks. Beyond that, we also collected the habitual diet data by asking the volunteer to fill out a short food frequency questionnaire [32] every 4 weeks. Our study demonstrated that after applying this enrichment protocol, significantly more fungal sequences could be captured with normal-depth shotgun sequencing. Moreover, with the dietary information, our study allowed us to link the habitual diet with microbial taxa and functions.

## Methods

### Fungal genomes collection

All species and strains used in this project were collected from the FunOMIC-T database [11] in order to use the marker genes. To analyze the ITS copy number, 260 fungal strains covered by seven species (*Aspergillus flavus, Candida albicans, Candida glabrata, Cryptococcus neoformans, Rhizopus oryzae, Rhodotorula mucilaginosa,* and *Saccharomyces cerevisiae*) were chosen, as explained in the Results section. For each of the selected strains, both its genome assembly and its corresponding raw shotgun sequencing reads were downloaded from the NCBI [33] or JGI [34] databases. Moreover, genome assemblies of the 14 most abundant species in the human healthy gut mycobiome were also downloaded to create in silico mock communities [11].

### Estimation of the ITS copy numbers

Two methods were used to estimate copy numbers of fungal ITS regions: Hidden Markov models (HMM) and mapping depth [16, 35]. To determine the ITS copy numbers using HMM, we created two HMMs respectively for predicting the flanking sequences of the two ends of the whole ITS region. These two HMMs are located separately in the large subunit (LSU) and small subunit (SSU) of the rRNA gene. For creating the HMM of the LSU, a total of 97 sequences in FASTA format were obtained from NCBI (Additional file 1: Data S1) which were then aligned using the “MUSCLE” tool [36] in Stockholm format. This alignment was then used to create the HMM profiles with the hmmbuild function in UNIX [37, 38]. For creating the HMM of the SSU, 100 sequences (Additional file 2: Data S2) were recovered and used through the same process as with the LSU. The resulting HMM profile had a length of 588 bp for LSU and 142 bp for SSU. With the obtained HMM profiles we searched the DNA homologies of the beginning and the ending of the ITS regions in each of the 260 genome assemblies mentioned above, then estimated the number of copies by using the nhmmer function. Then, we applied a filtering step to eliminate those matches that presented an E-value greater than 0.001 [39]. The copy number was then determined using an in-house Python script that evaluated the distance between the start and end of the ITS region. As most ITS lengths were reported in a range of 400 and 800 bp with an average of 550 bp [40, 41], the script accepted a distance between 400 and 800 bp. Moreover, if there was an HMM match for one end of the ITS but not the other, the script determined whether the prospective ITS sequence was at the end of a chromosome/scaffold, in which case the other end of the region could not be found, thus the script counted another copy. Once the copy number was determined for a genome, the complete ITS sequence was extracted by means of the BEDTools tool, which will be used in the mapping depth method mentioned below [42]. The resulting file contained three columns for each genome: genome ID, ITS copy number, and ITS sequence. Summary statistics were calculated for the seven species analyzed.

**Fig. 1:**
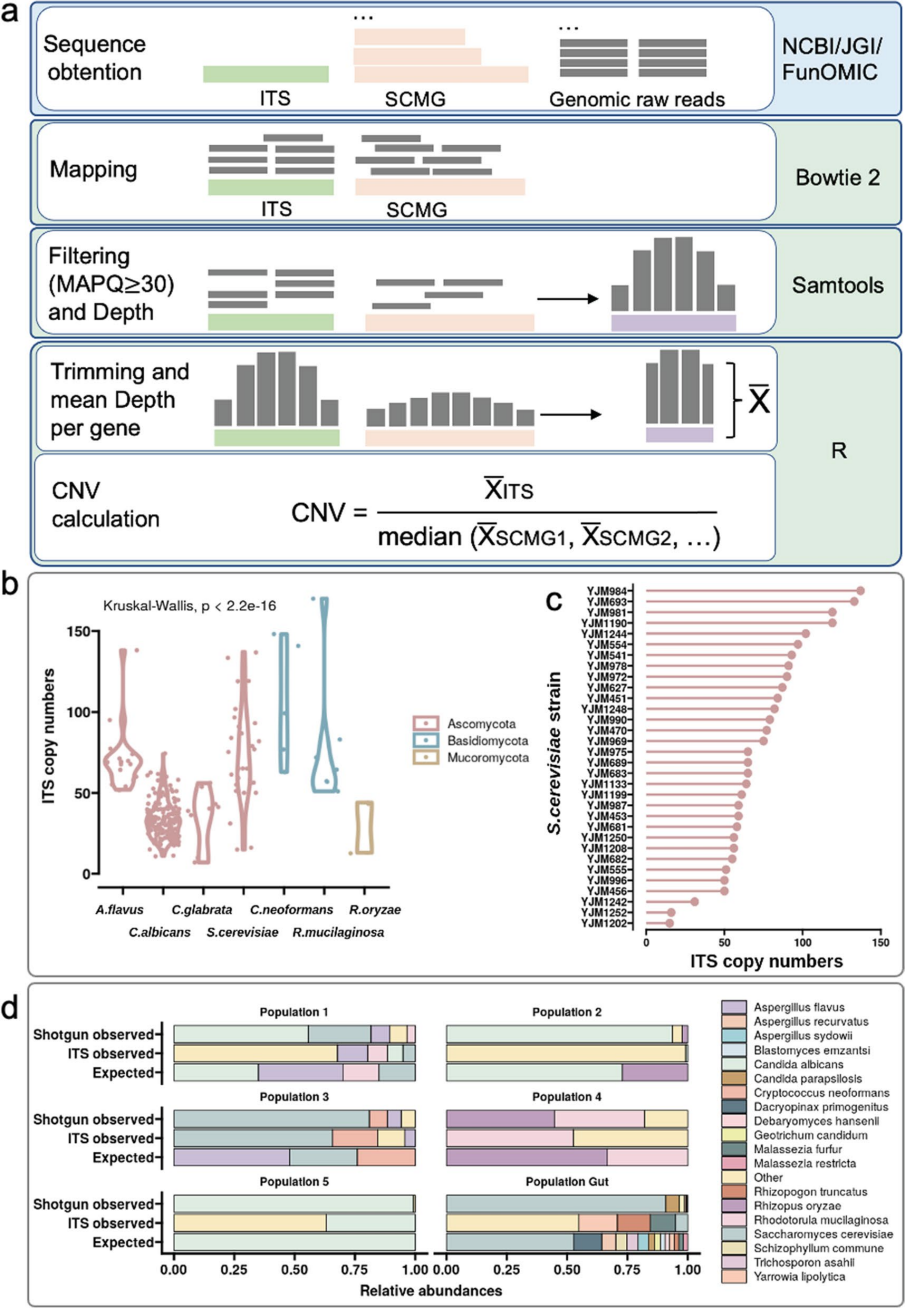
Shotgun sequencing provides higher accuracy than ITS sequencing in mycobiome profiling at the species level. **a** Workflow of the mapping depth approach to recover ITS copy numbers. **b** The distribution of CN-MD (*y* axis) of the ITS across the strains of the 7 analyzed species (*x* axis) (*n* = 260). **c** The intraspecific CN-MD of S. cerevisiae (*x* axis) for the 32 strains analyzed (*y* axis) (*n* = 32). **d** In silico comparison at species level between expected abundance (“Expected”) and observed abundance by using the shotgun method (“Shotgun observed”) and the ITS method (“ITS observed”)

To estimate CNV using the mapping depth approach, we calculated the ratio of the ITS depth against the single-copy marker genes’ depth [18]. The ITS sequence for each genome was obtained from the HMM method mentioned above while the single-copy marker genes were obtained from the FunOMIC-T database [11]. The raw reads of the 260 genomes previously downloaded from the NCBI and JGI were filtered and trimmed using Trimmomatic (version 0.36) with the default settings [43] to obtain reads with higher qualities. The filtered reads were then mapped respectively to the corresponding ITS sequence and the set of single-copy marker genes by using Bowtie2 [44]. Once the mapping was finished, samtools [45] was used to convert the resulting SAM file into a BAM file. Next, those reads mapped with a *q*-score inferior to 30 were filtered out and the depth at each base was calculated. The resulting file was then analyzed using R (version 4.0.2) where the mapping depth, which was determined as the mean depth of all the bases of each gene, was calculated and normalized by gene lengths. Prior to calculating the mean, the positions at the two ends (which present fewer reads and lead to a bias on the real mapping depth) were trimmed by deleting the first and last 50 bp, as done in the report of Lofgren et al. [16]. The copy number was finally estimated as the ratio of the mapping depth of the ITS region by the median of the mapping depths of all the single-copy marker genes. We used the median in order to avoid a bias of outlier single-copy genes that had a higher-than-usual mapping depth. The whole pipeline for this analysis is summarized in Fig. 1a.

To validate the above mapping pipeline, we selected 10 *S. cerevisiae* genome assemblies out of the 260 assemblies downloaded from NCBI. We recovered the ITS sequences from each of these assembled genomes using the CN-HMM method. Then we queried the ITS sequences in chromosome XII of each genome assembly to get the total number of hits, which is used as the reference ITS copy numbers. Then for each of the 10 assemblies, we generated 15 million sequence reads using the InSilicoSeq tool [46]. Those reads were then mapped to their respective ITS and single-copy fungal marker genes’ sequences using the read mapping pipeline to identify the copy number estimated by mapping depth (CN-MD). The Student *t* test was done to compare the CN-MD with the references, with a significant threshold *p* value < 0.05.

### In-silico comparison of ITS and shotgun methods

To compare the accuracy of the ITS and shotgun methods in detecting the relative abundance of fungi at strain, species, or genus level in environmental samples, genomes assemblies from 27 strains were used, for which the ITS sequences (by CN-HMM method) and copy numbers of ITS (CN-MD) were extracted. These were used as artificial genomes for creating mock communities. Five different in silico mock fungal communities were generated with randomly selected strains for each species. The strains and their randomly attributed abundances are shown in Additional file 3: Data S3. With the InSilicoSeq tool, we created 15 million reads [47] from the whole genomes (for the shotgun simulation) and another 15 million from the ITS sequences (for the ITS sequencing simulation) for each community (Additional file 4: Fig. S1). After obtaining the reads, the shotgun reads were mapped to the FunOMIC-T database, while the ITS reads were mapped to an in-house ITS database. The ITS database was created by integrating the UNITE [48] (version 8.2) and the RefSeq database [49] (data downloaded before 09/12/2020), as well as the sequences extracted from the HMM, in total of 96,388 sequences. The post-mapping processing was the same as the CN-MD pipeline. Then, an extra filtering step was taken: the filtering of those genes that presented less than 15 mapping depth, as they were possible off-target mappings and could be a cause of bias. This filtering was based on previous publications [50, 51], where various tests were undertaken to determine the optimal filtering value.

A lower mapping depth filter would result in the introduction of more off-target species while a higher filtering depth would reduce low-abundant but relevant species [50, 51]. Relative abundance for each mapping hit inside each mock population was calculated by dividing the mean depth of each hit by the sum of all mean depths. Then, the relative abundance of each species was retrieved by summing all the hits that corresponded to the relevant species; all other species were marked as off-target. The expected and observed relative abundances were then compared using R (v4.0.2), as described in the Statistical analysis section.

To further evaluate a more diverse fungal community and to mimic a gut microbiome sample, an additional mock community, consisting of the 14 most prevalent fungal species with their relative abundance found in healthy gut controls was created [11] (Additional file 3: Data S3).

### Fungal enrichment protocol

Centrifugation was used to separate fungal and bacterial cells based on their different cell sizes. Stoke’s law was used to estimate the centrifugation speed and time, in which D is the particle diameter (cm), η is the fluid viscosity (poise), Rf and Ro are the final and initial radius of rotation respectively (cm), ρp and ρf are the density of the particle and fluid respectively (g/ml), ω is the rotational velocity (radians/sec) and *t* is the required time for sedimentation from Ro to Rf (sec) (Eq. 1).

1 $$D=\left(\frac{18\eta l\eta \left(Rf/Ro\right)}{\left(\rho p-\rho f\right){\omega }^{2}t}\right)0.5$$

Briefly, 15 ml of 1 × PBS solution (Sigma-Aldrich Phosphate Buffered Saline Powder pH 7.4) was added to 500 mg fecal samples together with 10 2 mm glass beads (Merck KGaA glass beads 2 mm) to homogenize the feces into fecal suspension. The suspension was then passed through a 40-micron cell strainer (Clearline® cell strainers 40 µm blue color) to remove large-size undigested particles. We then centrifuged the filtered suspension for 3 min with 201 g using Eppendorf A-4-62. The supernatant was collected in a 50-ml falcon tube (deltalab EUROTUBO® 50 ml conical tubes) and the pellet was resuspended in 15 ml of 1 × PBS. The resuspended solution was centrifuged again for 3 min and 201 g with the same centrifuge to reduce the remaining number of bacterial cells from the fungi-enriched fraction, then the supernatant was collected and combined with the previous supernatant. We then resuspended the pellet with 1 ml of 1 × PBS and centrifuged it for 20 min at 10,000 × *g* using an Eppendorf Centrifuge 5427R to collect the pellet containing the enriched fungal cell fraction. The combined supernatant was centrifuged parallelly in Fiberlite™ F14-6 × 250LE for 30 min at 10,000 × *g*, the pellet was collected as the enriched bacterial cell fraction (Additional file 5: Fig. S2).

### Collection and processing of habitual diet information

Six volunteers, free of diagnosed diseases, were recruited between August 2021 and October 2021 by disseminating an announcement. During 2 months, each volunteer filled a short Frequency Food Questionnaire (sFFQ) [32], in total 12 sFFQs were collected.

Nutrients were adjusted by energy using the residual method [52] to control the confounding effect of calories. We then used the Wilcoxon test and the intraclass correlation coefficient (ICC) [52, 53] to evaluate the reproducibility of the sFFQ by comparing both the energy-adjusted nutrient data and the food groups extracted from the sFFQ administered on two-time points.

### Sample collection and DNA extraction

Each of the above-mentioned volunteers donated one fecal sample per week for 2 months, in total 48 fecal samples were collected. The fecal samples were frozen immediately at – 20 ℃ then transferred to – 80 ℃ within the month. For each of the 48 samples, two aliquots of 500 mg were taken, one was used directly for the DNA extraction, and the other was separated into the fungal enriched partition and enriched bacterial partition by applying the fungal enrichment protocol before the DNA extraction. Thus, three partitions per sample (enriched in fungi, enriched in bacteria, and control without enrichment), in total 143 samples (volunteer No. 4 did not provide enough feces for time point 1 so only one aliquot was obtained for getting the enriched fungal and bacterial partitions) were processed for genomic DNA extraction as previously described [54].

### Shotgun metagenomic sequencing and profiling

Shotgun metagenomic sequencing was applied to the 143 extracted genomic DNA using the Illumina Novaseq 6000 platform. The average reading depth was 6.45 Gbp. For each of the sequencing samples, we used the KneadData v0.7.7-alpha tool (https://huttenhower.sph.harvard.edu/kneaddata/) for trimming out low-quality reads and decontaminating human sequences. Then, an unpublished updated version of the FunOMIC database that contains 2 million single-copy marker genes and 21 million protein sequences extracted from more than 3000 fungal species [11] was used for getting the raw reads of taxonomic and functional mycobiome profiling. The Original version of this database is available at https://manichanh.vhir.org/funomic/, the complete release of the updated version is under preparation. Then the raw reads were normalized with the TMM method [55, 56] using the R package “edgeR”. The MetaPhlAn v3.0.9 and the HUMAnN v3.0 [57] (https://huttenhower.sph.harvard.edu/humann/) were used respectively for the taxonomic and functional prokaryotic microbiome profiling. The functional profiling output by HUMAnN was annotated using MetaCyc pathway database [58] while that of FunOMIC was using the KEGG pathway database [59]. To make the annotations consistent, we regrouped the prokaryotic functional profiling into KEGG annotation style by using the function “humann_regroup_table” embedded in HUMAnN and the function “keggLink” under R package “KEGGREST” (Dan Tenenbaum and Bioconductor Package Maintainer (2021). KEGGREST: Client-side REST access to the Kyoto Encyclopedia of Genes and Genomes (KEGG).).

### Keystone species analysis

The network was constructed based on the species-level SparCC correlation matrix measured using the SparCC tool which uses logarithmically scaled variances to calculate correlations between species [60]. We inferred and removed the indirect effects from the observed correlation matrix by using the network deconvolution algorithm as previously proposed [61, 62]. Then based on the random matrix theory (RMT), we determined a threshold of rho = 0.78. All correlations that had an absolute value of less than 0.78 were discarded [63]. The *p* values for all the correlations were adjusted using the Benjamini and Hochberg false discovery rate (FDR), and a cutoff of FDR = 0.001 was applied to remove the non-relevant correlations. The resulting correlation matrix was then used to construct the network using the R package “igraph” [64]. “The igraph software package for complex network research.” InterJournal, Complex Systems, 1695. https://igraph.org.). After network construction, the topological indices, including the degree, betweenness centrality, and closeness centrality of each node, were calculated by using functions developed in igraph.

### Statistical analysis

To compare the ITS and the shotgun approaches, weighted UniFrac distances [65] were calculated using the phyloseq package [66]. Distances were compared between methods using a Student *t* test [67], as the values belonged to a normal distribution, proved beforehand by doing a Shapiro test [68]. Spearman correlations of dietary data or metadata with microbiome alpha diversities or microbiome taxonomic and functional compositions were computed using the cor.test from the stats R package (v4.0.2). The p-values for all the correlations were adjusted using the Benjamini and Hochberg FDR. We considered significant correlations with an FDR < 0.05. In the heatmaps for partial correlations, the asterisk indicates that the correlation index for the corresponding species metadata pair is significant.

## Results

### Shotgun metagenomics sequencing provides higher accuracy than ITS amplicon sequencing in mycobiome profiling at the species level

#### Inaccuracy in the genome assembly of the ribosomal region of the fungal genomes

To analyze the copy number variability of the ITS region, we recovered 260 assembled fungal genomes covering seven fungal species, known to be relevant in human microbiome studies: *Saccharomyces cerevisiae*, *Aspergillus flavus*, *Candida albicans*, *Candida glabrata*, *Cryptococcus neoformans*, *Rhodotorula mucilaginosa*, and *Rhizopus oryzae* [11]. From each of these genomes, we calculated the copy number of the ITS regions using Hidden Markov Models (CN-HMM) and an in-house bioinformatic pipeline. All species, except *S. cerevisiae*, presented a very low copy number of ITS (average of 2) and this did not vary much across species. This observation is not in agreement with a previous study that reported a high number of ITS copies, ranging from 14 to 1442 [16] (Additional file 6: Data S4). Furthermore, we recovered only one copy for most of the *C. albicans* strains. However, *C. albicans* is well-studied and has been shown to carry 21 to 200 copy numbers (CN) per genome [69–71]. The other five species presented also a very low mean CN-HMM value (Additional file 6: Data S4). Together, these results suggest an inaccuracy in the assembly of fungal genomes, at least in the region of the ribosomal genes.

#### High inter- and intra-species variability in the ITS copy number

Genomes that contain many repetitive sequences have usually been difficult to assemble when short sequence reads have been generated. Indeed, during assembly, repetitive regions such as the ITS regions are algorithmically collapsed into only a few sequences due to their similarity, leading to a potential bias in the CN-HMM estimation. To circumvent the bias introduced by the incomplete fungal genomes, we used a mapping depth method for estimating ITS copy numbers (CN-MD) (Fig. 1a), for which more details are described in the Methods section. Before estimating the ITS copy number, we first validated the mapping pipeline. Ten genome assemblies of *S. cerevisiae* were selected for this validation, and their ITS copy numbers were retrieved from the NCBI nucleotide database to work as the expected ITS copy number. At the same time, we generated 15 million simulated shotgun sequencing reads of the 10 assemblies using the InSilicoSeq tool [46]. The simulated reads were then used as the input of the mapping pipeline for calculating the CN-MDs for each of the genomes. At last, the calculated CN-MDs were compared with the reference copy numbers, by applying the Student t-test. The comparison between the two values did not show significant differences (Additional file 7: Data S5, *p* value = 0.28), which indicates that the pipeline could reliably recover the expected ITS copy numbers from whole genome shotgun sequencing reads.

Next, we applied the mapping pipeline to estimate the CN-MDs of the 260 assembled fungal genomes using their shotgun sequencing reads downloaded from NCBI or JGI. The resulting CN-MD ranged from 7 to 170, with an average of 60 (Additional file 8: Data S6). We observed that both the intra- and inter-species variability was high for the ITS copy numbers of the analyzed genomes which cover seven species and three phyla (Fig. 1b). The copy numbers of the ITS region of the 32 collected *S. cerevisiae* strains were widely distributed, ranging from 15 to 137 (Fig. 1c) and those of the 182 *C. albicans* strains varied from 11 to 74. The variance between *C. albicans* and *S. cerevisiae* was significantly different (*p* value = 8e−10; Levene’s test). These findings indicate that possible bias could be introduced when profiling the fungal community by using ITS amplicons without normalizing by the actual strain level ITS copy numbers.

#### Shotgun data are more accurate than ITS data for taxonomic profiling at the species level

To compare the accuracy of the species-level mycobiome profiling generated by ITS sequencing and shotgun sequencing, we created in silico mock communities with different groups of fungal species. A total of 27 artificial fungal genomes with known ITS copy numbers were used to create five *in-silico* mock communities. We randomly generated relative abundances for the species in each of the five communities (Additional file 3: Data S3). The artificial genomes and their ITS sequences were used to simulate the shotgun sequencing reads and the ITS sequencing reads, respectively. An additional mock community mimicking the gut mycobiome was also created using the 14 most abundant gut fungal species and their observed relative abundance based on a previous study [11] (Additional file 3: Data S3). The annotations for both sequencing methods were done using QIIME2 and FunOMIC pipelines for ITS and shotgun reads, respectively.

To compare the efficiency of the two methods in performing taxonomic profiling, we calculated weighted UniFrac metrics, which then allowed us to test whether phylogenetic lineages between samples were significantly different. The metrics were calculated between the observed taxonomic profilings generated from both sequencing methods and the fixed relative abundances of the six mock communities, at the species and genus levels. At the genus level, the results showed that the two methods were not significantly different (*p* value = 0.623, Student *t* test). The ITS method exhibited a mean distance of 0.263 and the shotgun method exhibited a mean distance of 0.213 (Additional file 9: Data S7), which indicates that both methods showed similar accuracy in taxonomic profiling at the genus level. However, at the species level, the mapping results (Fig. 1d) showed that the two methods differed significantly (*p* value = 0.005, Student *t*-test). The ITS method exhibited a mean distance of 0.616 and the shotgun method a mean distance of 0.237 (Additional file 9: Data S7, Additional file 10: Fig. S3), indicating that the shotgun method was able to recover the expected fungal community compositions more reliably at the species level. The same analysis at the strain level was also employed, however, the results revealed that neither shotgun nor ITS sequencing was accurate enough to detect the specific strains.

### A fungal enrichment protocol effectively concentrates fungal cells in human fecal samples

As demonstrated by previous studies [11, 26], the proportion of fungal sequences obtained upon shotgun sequencing of DNA prepared from human fecal samples consists of less than 0.08% of the total sequences, which limits the accuracy of the recovered fungal community composition results if the sequencing depth is not high enough. However, the cost of deep shotgun sequencing is still not easily affordable by all researchers. We thus proposed an enrichment protocol based on a series of centrifugations to separate fungal and bacterial cells prior to the regular DNA extraction method.

**Fig. 2:**
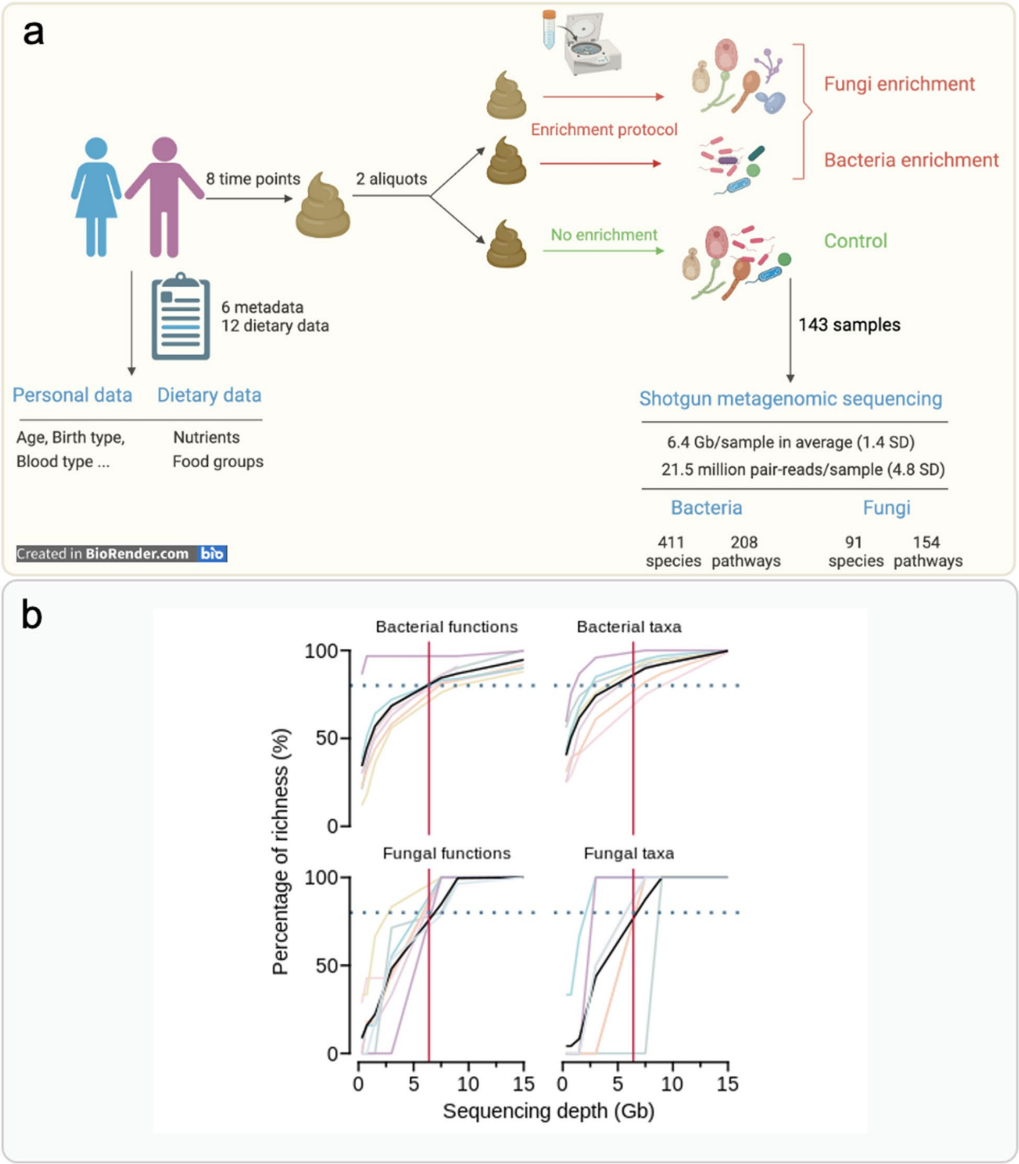
Study design and quality of shotgun sequencing. **a** Design and workflow of this study. **b** Rarefaction curves of the shotgun sequencing, *x*-axis represents the depth of sequencing, *y*-axis represents the percentage of richness. The rows of the panel are different microbial communities, and the columns of the panel represent the taxonomic or functional level richness. The black solid line is the average percentage of the richness of the 8 samples at the specific depth of subsampling. The red vertical solid lines represent the average sequencing depth 6.4 Gb, the blue horizontal dotted lines represent the threshold of 80% richness

To evaluate the practical efficiency of this enrichment protocol, we collected fecal samples from six healthy volunteers that included three females and three males. Each of the volunteers donated their fecal samples weekly during an 8-week span, making up a batch of 48 fecal samples. Then, for each of the 48 samples, two aliquots of 500 mg were kept, from which one aliquot underwent the enrichment protocol to be separated into a fungal enriched partition and a bacterial enriched partition, while the other aliquot did not pass any further operation and was used as the unenriched control. Finally, a total of 143 partitions (one of the volunteers did not provide enough feces for two aliquots) of fecal samples were sent for shotgun sequencing using the Illumina Novaseq 6000 platform (Fig. 2a). The sequencing provided an average of 6.4 Gb, and 21.5 million pair reads which are comparable with other studies using shotgun sequencing [5, 72, 73]. Next, we annotated the bacterial and fungal communities for all 143 samples using HUMANn [57] and FunOMIC pipelines [11]. A total of 411 bacterial species and 208 KEGG pathways were found in the bacterial community, and 91 fungal species and 154 KEGG pathways were found in the fungal community. To assess whether the sequencing depth was sufficient to recover the majority of both fungal and bacterial richness, we selected eight samples that had the highest number of Gb to perform rarefaction curves. Each sample was subsampled and annotated with a gradient of sequencing depths. With the cutoff of the 6.4 Gb, around 80% of fungal taxonomic richness, more than 70% of fungal functional richness, 100% of bacterial richness, and almost 100% of bacterial functional richness were recovered, showing that our shotgun sequencing run was able to capture most of the microbiome information (Fig. 2b, Additional file 11: Fig. S4). The plateau was reached at 7.5 Gb for fungal taxonomy, 15 Gb for fungal functions, and 6.7 Gb for bacterial functions. Together, these results showed that a sequencing depth of 15 Gb would allow the capture of the entire bacterial and fungal communities.

**Fig. 3:**
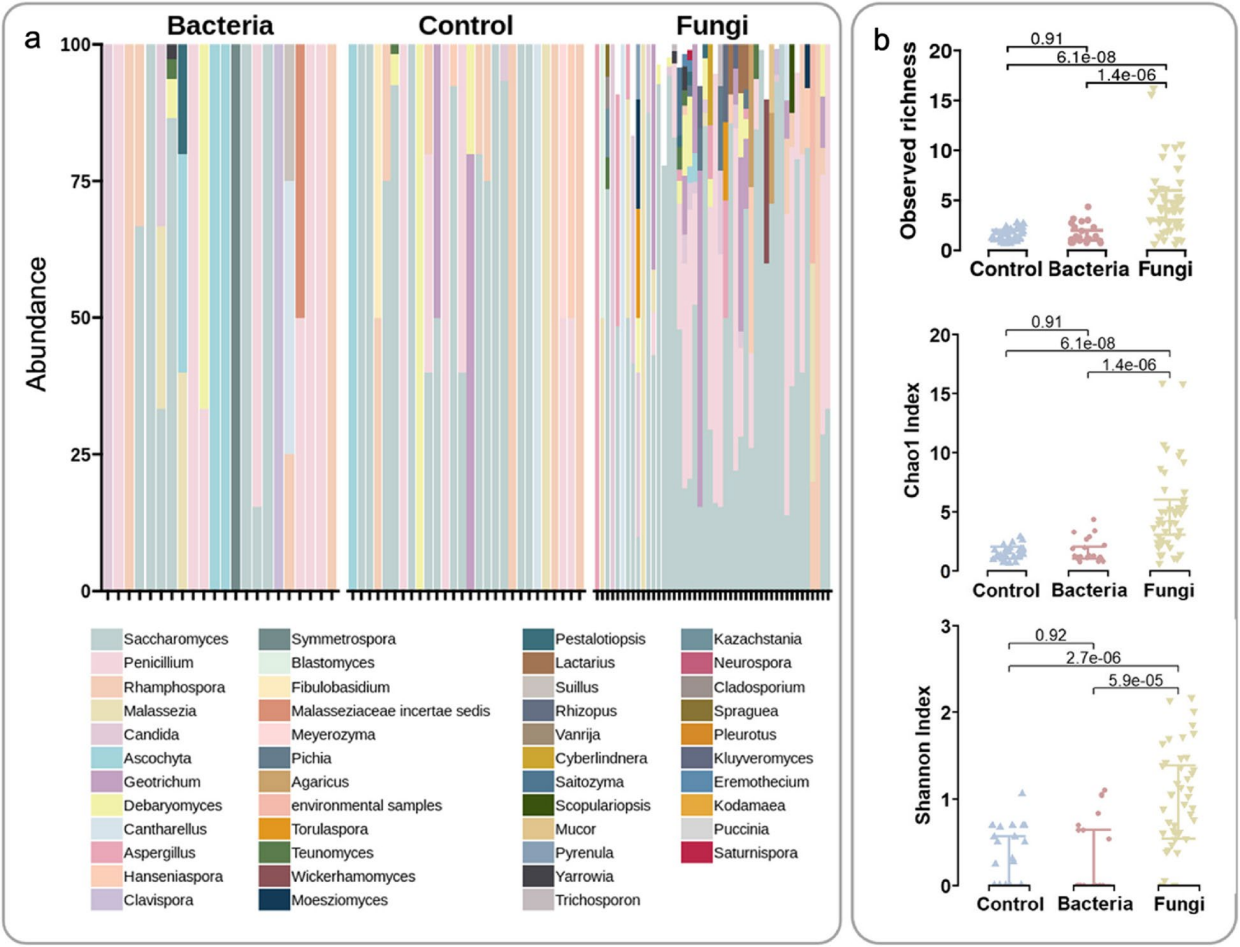
Enrichment efficiency in fungi. **a** The genus-level taxa bar plot of the fungal community compositions in bacterial, control, and fungal partitions. **b** Boxplots of the species-level fungal community alpha diversities (observed species, Shannon, and Chao1 indices) in control, bacterial, and fungal partitions (*n* = 96), ordered by their mean from smallest to largest (left to right)

Then, we mapped each of the 143 samples to fungal and bacterial databases [11, 57] to calculate the enrichment efficiencies and meanwhile get their fungal and bacterial, taxonomic, and functional profiles. Notably, the fungal profiling was annotated with the unpublished updated version of the FunOMIC database that contains 2 million single-copy marker genes and 21 million protein sequences extracted from more than 3000 fungal species. In total, we have detected fungi in 96 samples out of 143 (67%). We observed that by applying the enrichment protocol, the proportion of samples that have fungi detected increased from 58.3 (28 out of 48) to 95.8% (46 out of 48). We then calculated the ratio of the reads that were mapped to the fungal database against the reads that were mapped to the bacterial database for all 143 samples. Then, we used this ratio in the fungal partition and divided by this ratio into their corresponding control partitions to estimate the extent to which the fungal sequences have been enriched. The ratio increased on average 18.47 times (ranging from 0.07 to 235) after applying the enrichment protocol, and the fungal alpha diversity in fungal enriched partitions was found significantly higher than in both bacterial (*q* = 4.3e−5 Shannon index, *q* = 2e−7 Chao1 index) and control partitions (*q* = 5.2e−5 Shannon index, *q* = 3.7e−6 Chao1 index) (Fig. 3a, b).

Similar but less significant results were found in the bacterial partitions (Additional file 12: Fig. S5a, b). Genus-level taxa bar plot of the fungal and bacterial communities grouped by different time points can also be found in Additional file 13: Fig. S6. Since bacterial reads were still present in the fungal partitions, they were merged with those in their corresponding bacterial partitions. After applying a paired Wilcoxon test between the bacterial alpha diversities before and after merging, we found the Chao1 index after merging had a trend of being higher than that of before merging (*p* = 0.06, Additional file 12: Fig. S5c). This result was not observed for the fungal alpha diversity. For the purpose of capturing more information, in the subsequent analysis, the merged bacterial microbiome profiling was used to represent the bacterial community, and the fungal microbiome profiling in fungal partitions was used to represent the fungal community in each sample.

### Keystone bacterial and fungal species in the human gut

**Fig. 4:**
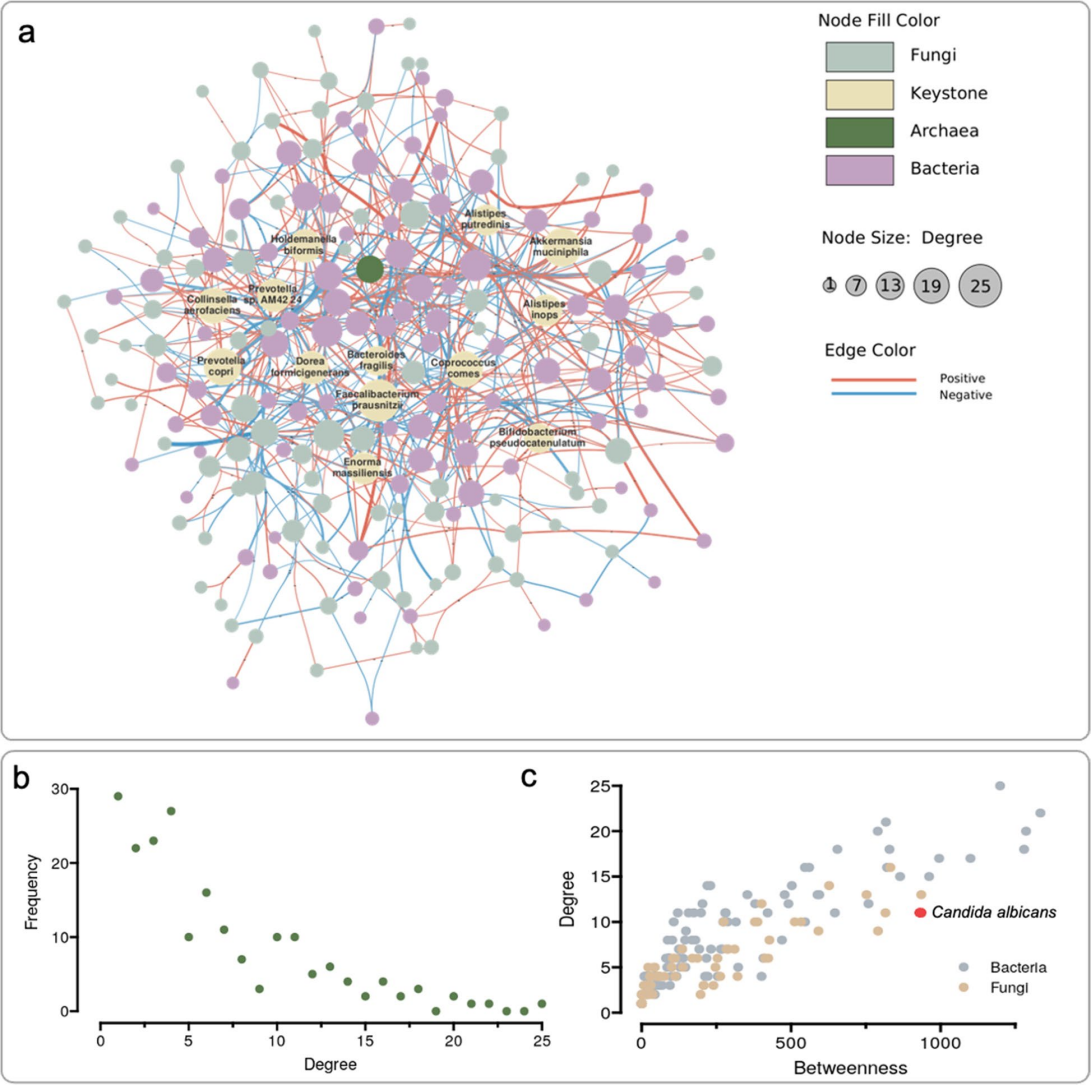
Inter-kingdom network and keystone species. **a** Network of the SparCC correlation between the fungal and bacterial taxonomic composition at the species level (FDR < 0.01). Each node shows a unique microbial species, each edge showing the SparCC correlation between the two nodes linked. The edges connecting the nodes represent significant correlations (FDR < 0.001). The color of the nodes represents the kingdom of the specific species and highlights the keystone species. The size of the nodes represents the node degrees for each node. The color of the edges represents the symbol of the correlation, and the width of the edges represents the intensity of the correlation. **b** Degree distribution of the network following a scale-free distribution. **c** Candida albicans, one of the fungal species that has a high node degree and the highest betweenness centrality

To determine the keystone species in the human gut microbiome, we constructed networks based on the SparCC correlation matrix and the corresponding BH-adjusted P-values matrix. In each network, the nodes represent the microbial species that were included in this network, and the edges connecting the nodes represent the significant inter-kingdom correlations (FDR < 0.001). This network captured 625 associations among 199 microbial species which includes 111 bacterial species, 87 fungal species, and 1 Archaea species (Fig. 4a). Among the 625 associations 349 were positive and 276 negative associations. This network consisted of only one large connected group (199 out of 199 microbial species (100%)). The global network had an average node degree (number of edges adjacent to the node) of 6.28 (7.66 for bacteria and 4.5 for fungi), and it perfectly followed a scale-free degree distribution (power law) (Fig. 4b), indicating that most nodes had low-degree values, and only a few nodes had the highest degree values, which are often called “hubs”, and are thought to serve specific purposes in the networks. In this network, “hubs” are microbial species that have a much higher number of correlations among all the species, indicating that they are more active within the gut microbiome context.

Two fungal species *Eremothecium sinecaudum* and *Candida albicans*, were found to have the highest betweenness centrality (the number of shortest paths going through a node) (945), and high node degree (11) among all the fungal species in this network (Fig. 4c), suggesting a critical role in the gut microbial community. Among them, *C. albicans* is a known fungal pathogen [74], was found to form in the gut microbiome seven cross-domain associations with *Ruminococcus gnavus, Firmicutes bacterium CAG 110, Streptococcus salivarius, Holdemanella biformis, Eubacterium sp CAG 274, Proteobacteria bacterium CAG 139,* and *Alistipes inops*. From the species analysis (using betweenness centrality and node degree), we identified one fungal species and 13 bacterial species as potential gut keystone species (Table 1) as they were the species that appeared in both the list of the top 20 highest node degree and the top 20 highest betweenness centrality (top 20).

**Table 1:** Keystone microbial species in the human gut

**Keystone species**	**Betweenness centrality**	**Node degree**
*Faecalibacterium prausnitzii*	1199.25	25
*Bacteroides fragilis*	864.43	15
*Enorma massiliensis*	1100.07	17
*Alistipes inops*	995.29	17
*Prevotella sp AM42 24*	654.86	18
*Collinsella aerofaciens*	789.85	20
*Akkermansia muciniphila*	1334.21	22
*Alistipes putredinis*	821.04	16
*Dorea formicigenerans*	829.1	18
*Coprococcus comes*	1286.18	20
*Holdemanella biformis*	1279.61	18
*Prevotella copri*	817.2	21
*Bifidobacterium pseudocatenulatum*	961.73	15
*Debaryomyces hansenii*	832.15	16

### Short-term dynamics of the human gut microbiome

**Fig. 5:**
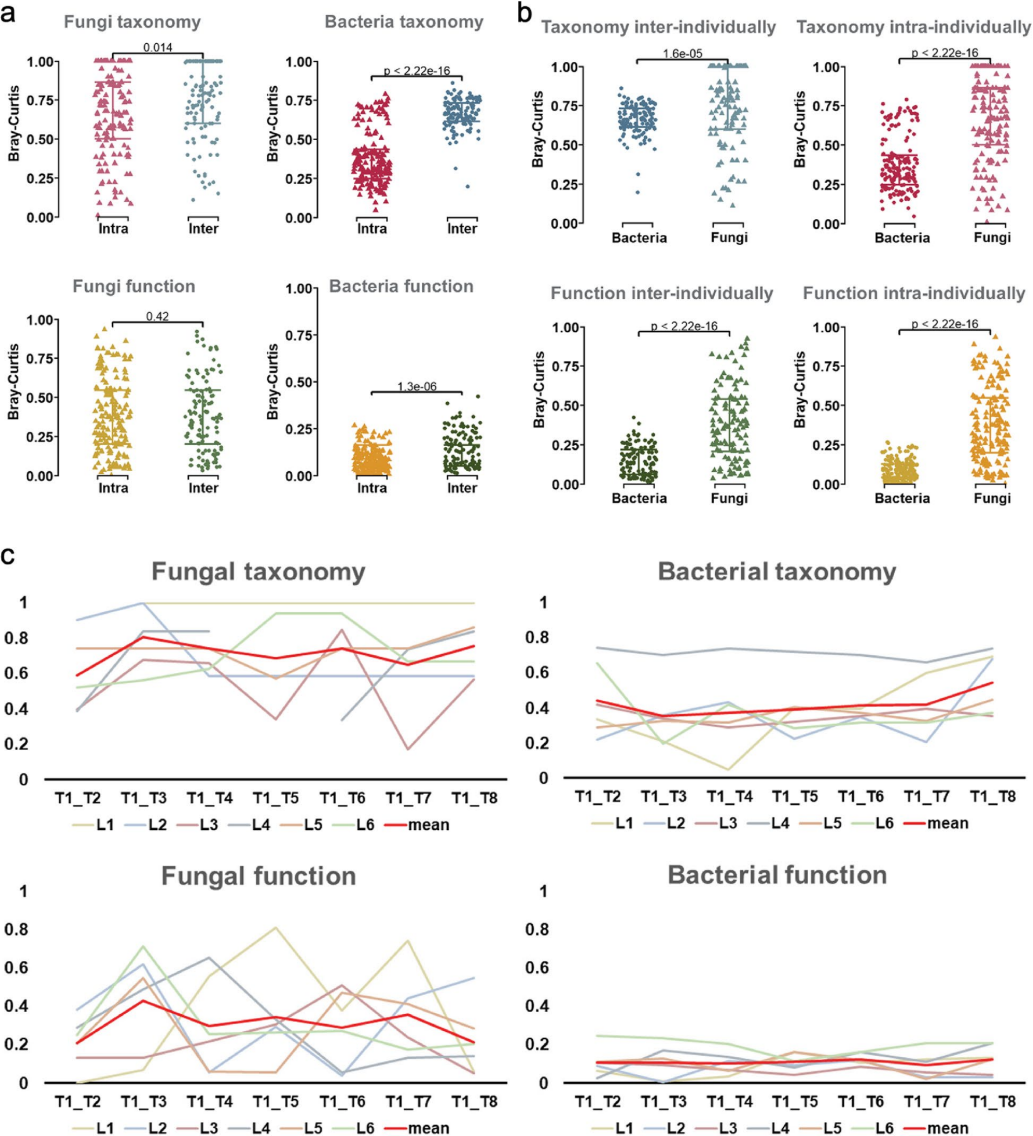
Dynamics of the human gut microbiome. **a** Intra- and inter-individual beta diversity (Bray-Curtis) in fungal and bacterial communities at taxonomic and functional levels. **b** Comparison of the beta diversities between fungal and bacterial communities intra- and inter-individually at taxonomic and functional levels. **c** Dynamics of fungal and bacterial communities at taxonomic and functional levels. The *x*-axis represents different time points, the *y-*axis represents Bray-Curtis values

To determine the intra- and inter-individual variability of the volunteers’ gut microbiome, we measured the pairwise dissimilarities using the Bray-Curtis dissimilarity values between longitudinal samples donated by the same volunteer and between samples donated by different volunteers for both fungal and bacterial microbiomes. The results revealed that both bacterial and fungal communities exhibited higher inter-individual than intra-individual dissimilarities (Fig. 5a), while this difference was significantly more pronounced in the bacterial community. We then compared the dissimilarity between fungal and bacterial communities; the variabilities in the fungal community were significantly higher than in the bacterial community (Fig. 5b).

Then, to investigate the stability of the gut microbiome over time, we considered the first time point as a baseline, and for each of the individuals, we measured the Bray-Curtis dissimilarities of other time points against the baseline. In both taxonomy and function, despite the high degree of short-term longitudinal change in both communities, we found that the fungal community displayed increased dynamics as compared to the bacterial community (Fig. 5c). Notably, the mean Bray-Curtis values calculated from data of the six individuals were significantly higher for the fungal microbiome than bacterial microbiome (Wilcoxon, *p* < 0.01 for both taxonomy and functions). To determine whether dietary changes drove the dynamics of the gut microbiome, we correlated the pairwise Bray-Curtis dissimilarity of the microbiome (fungal and bacterial, taxonomy and function) with the pairwise Bray-Curtis dissimilarity of dietary data (nutrient macromolecules and food groups). However, no significant correlations were found, which may indicate the absence of an effect of the diet on the microbiome composition and function at the global level but does not exclude the effect of specific food groups or nutrients.

### Microbial diversity and composition are associated with habitual diet

**Fig. 6:**
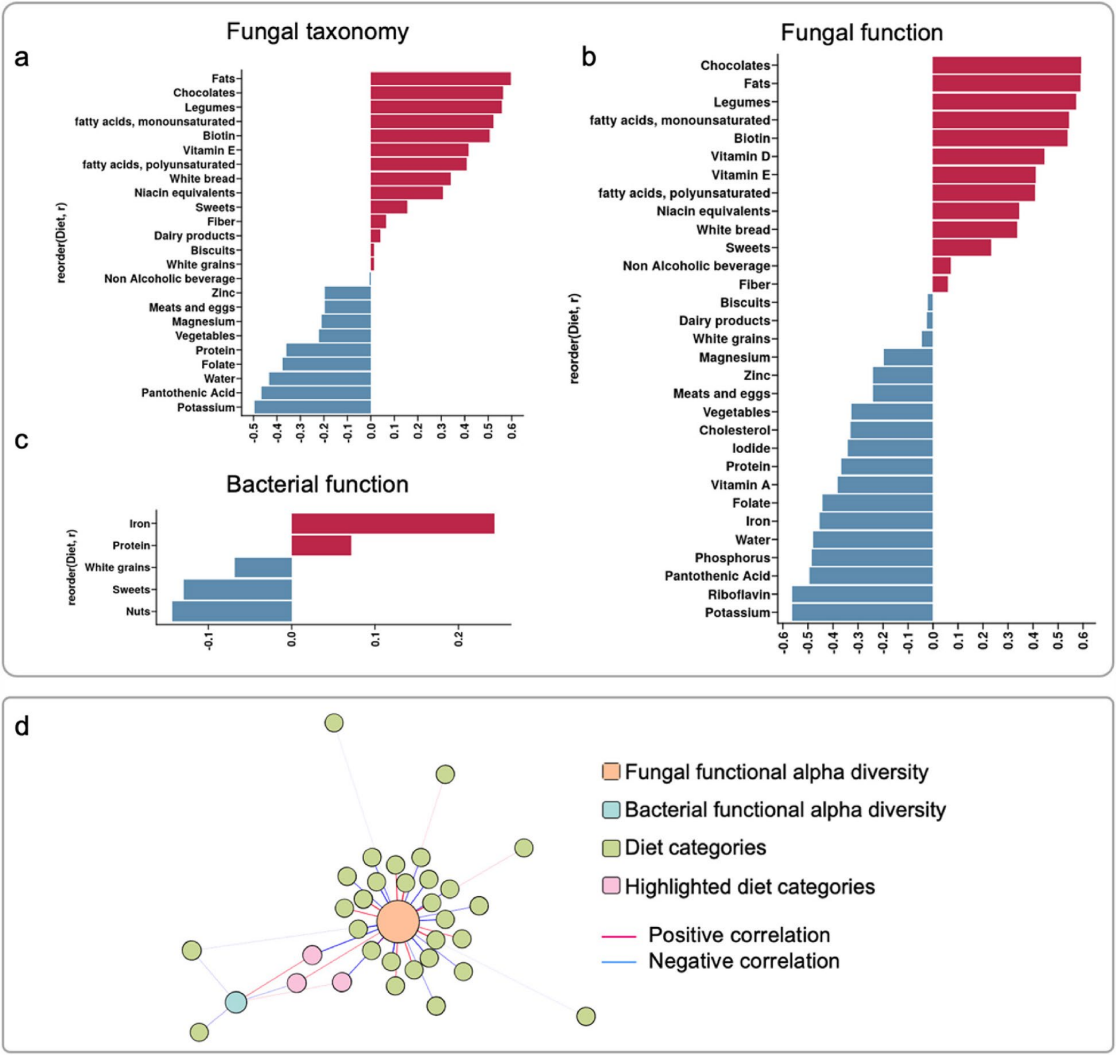
Microbial taxonomic and functional alpha diversities are associated with habitual diet. **a** Significant (FDR < 0.05) Spearman correlations found between the fungal taxonomic alpha diversity and diet categories. The x-axis is the value of the correlation coefficient, the y-axis is the name of the diet categories. **b** Significant (FDR < 0.05) Spearman correlations found between the fungal functional alpha diversity and diet categories. The x-axis is the value of the correlation coefficient, the y-axis is the name of the diet categories. **c** Significant (FDR < 0.05) Spearman correlations were found between the bacterial functional alpha diversity and diet categories. The* x*-axis is the value of the correlation coefficient, the *y*-axis is the name of the diet categories. **d** Network of the fungal functional alpha diversity, bacterial functional alpha diversity, and diet categories detected to be significantly (FDR < 0.01) correlated with them. The *r* values are labeled for the overlapped diet categories. The edges are the Spearman correlation coefficients between the two nodes linked. The color of the edges represents the symbol of the correlation. The size of the nodes represents the node degree values

We then assessed the correlation between habitual diet (nutrients and food groups) and the alpha diversity of the human gut microbiome to get a broad view of how habitual diet could modulate microbial communities. Interestingly, using the Spearman correlation coefficient, we detected 21 significant associations (FDR < 0.05) with fungal taxa while no significant associations were found with bacterial taxa (Fig. 6a). Furthermore, 31 significant associations were found with fungal functions, and five significant associations with bacterial functions (Fig. 6b, c). The overlapped associations found with fungal taxa and fungal function were all consistent, whereas the overlapped associations detected with fungal function and bacterial function were all opposite, indicating that fungal and bacterial communities are likely to act competitively for some dietary products (Fig. 6d).

**Fig. 7:**
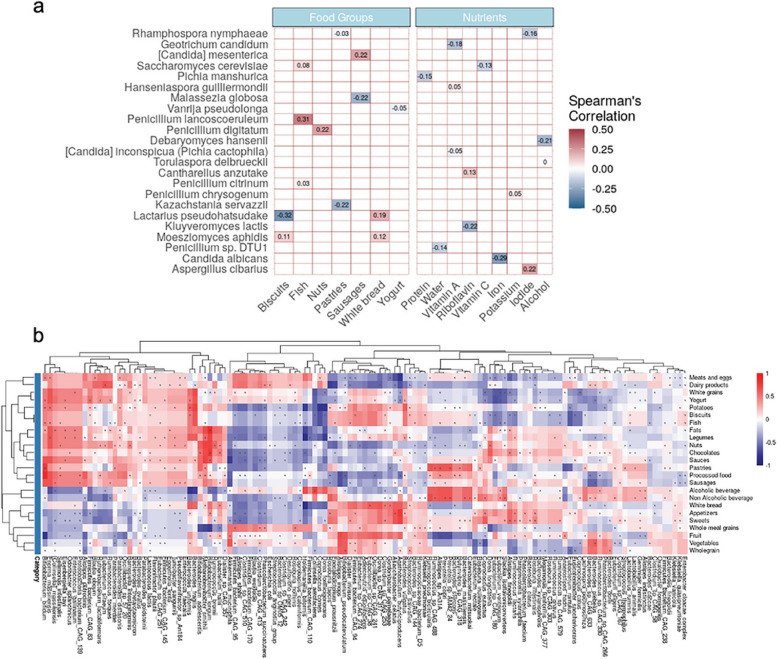
Microbial taxonomic compositions are associated with habitual diet. **a** Heatmap of all the detected significant correlations between fungal taxonomic compositions and diet categories. **b** Heatmap of all the detected significant correlations between bacterial taxonomic compositions and diet categories. The asterisk indicates that the correlation index for the corresponding species metadata pair is significant. For better visualization, this plot with higher resolutions can be found in Additional file 14: Fig. S7

Next, we calculated the Spearman correlation coefficients between a habitual diet, specific gut microbiome components, and functional pathways. We found 23 fungal species were significantly correlated with one or more dietary categories. Among them, the strongest correlations were *Lactarius pseudohatsudake* with biscuits (rho =  −0.32, FDR = 0.027), *Penicillium lancoscoeruleum* with fish (rho = 0.31, FDR = 0.027), *Candida albicans* with iron (rho =  −0.29, FDR = 0.038) (Fig. 7a). More significant correlations were detected in the bacterial community. At a broad level, we found three apparent groups of species clustered to a group of foods mainly classified as related to more animal-based foods (fish, sauces, sausages, processed food, dairy products) and two others related to less animal-based foods (fruit, vegetables) (Fig. 7b). Similar but less obvious groupings were also found when correlating habitual diet with microbial functions (Additional file 15: Fig. S8; Additional file 16: Fig. S9b).

## Discussion

In this study, we took advantage of our recent implementation of the most comprehensive fungal databases that contain 2 million single-copy marker genes and 21 million protein sequences extracted from more than 3000 fungal species [11] as well as a fungal community enrichment protocol in order to propose a robust approach integrating bacterial and fungal shotgun metagenomics data and characterize the human gut microbiome and its modulation by dietary components.

First, a series of in silico simulations led us to conclude that shotgun sequencing provides higher accuracy than ITS sequencing in mycobiome profiling at the species level. Indeed, we have evidenced the high intra- and interspecific variabilities of the fungal ITS region at the strain level. Similar results have also been reported previously, where 14 to 1442 ITS copies were found in 91 fungal taxa [16], 22 to 227 copies across the 788 *S.cerevisiae* isolates [18], and 38 to 91 18S copies in 8 *Aspergillus fumigatus* strains [14]. Given that the highest resolution of the ITS region barely reaches the species level [75], normalization of the ITS counts cannot reach the strain level, thus, accurate quantification of the fungal community in a complex ecology is impossible. Shotgun metagenomic sequencing plus the annotation using fungal single-copy marker genes offers an alternative. Our comparison of the performance of the ITS sequencing and the shotgun sequencing with in silico simulated mock community reads has supported this hypothesis. Though ITS sequencing is always considered a more cost-effective approach in analyzing fungal microbiomes, with the rapid development of next-generation sequencing technologies, the cost of shotgun sequencing has dropped to a more affordable level, taking into account that shotgun sequencing skips the amplification and amplicon purification steps. In sum, the total cost of both sequencing methods can differ slightly, while shotgun sequencing is able to capture more information including the functions of the fungal communities, and the taxonomy and functions of the bacterial communities. Thus, we strongly recommend researchers in this field switch to the usage of shotgun metagenomic sequencing when studying the fungal microbiome in the future.

To reduce the bias introduced by the low proportion of fungal cells in human fecal samples, we proposed a fungal enrichment protocol that effectively concentrated fungal cells. This protocol successfully increased the detected fungal counts and richness based on the separation provided by the centrifugation method. A membrane filter approach was also tested in parallel to assess the centrifugation method. Both methods utilized the nature that most bacterial cells are smaller than fungal cells. Several cellulose nitrate filters with different pore sizes (0.65 microns, 3 microns, and 5 microns) were used individually to intercept the fungal cells and release the bacterial cells. Nonetheless, the membrane method was hard to implement, for the intercept fungal cells and other impurities that failed to be removed immediately blocked the pores.

The network analysis has suggested candidate keystone microbial species including 13 bacterial species and 1 fungal species in the human gut environment. The only fungus identified as the keystone species, *Debaryomyces hansenii*, has been implicated as a fungus that is found in Crohn’s disease tissue and can lead to dysregulated healing. Crohn’s disease is usually characterized by the dysbiosis of the gut microbiome, bacterial species correlating with *D.hansenii* might play crucial roles in keeping the gut microbiome in a healthy balance. Among the bacterial species correlating with *D.hansenii*, *Faecalibacterium prausnitzii*, *Enorma massiliensis, Collinsella aerofaciens*, and *Prevotella copri* were also identified as the keystone species. *F. prausnitzii* is well known as one of the most abundant and important bacterial species in the human gut, it is also an important butyrate and other short-chain fatty acid producer in the gut microbiome.

We found that the mycobiome was much more dynamic than the bacterial community at the taxonomic and functional levels, which is consistent with the results found in other studies [73, 76], indicating that the fungal compositions in human gut shift rapidly instead of level off to a stable status such as the bacterial community. Since the habitual diet was found to have an influence on the composition of the fungal microbiome in both human and mice models [77–81], we sought the relationship between the dynamics of habitual diet and the dynamics of the gut microbiome. Although the microbiome changes were not driven by global dietary changes, we showed that microbial diversity, composition, and functions were associated with habitual diet composition. We have found that bacterial alpha diversity and fungal alpha diversity were oppositely correlated with three diet categories, sweets, protein, and iron. The level of iron in the habitual diet was found to negatively correlate with the fungal functional alpha diversity. To the best of our knowledge, this is the first demonstration of the effect of iron on the fungal functional alpha diversity, though some studies have discussed that high iron levels promote the growth of specific fungal species [82]. Our study indicates a potential competitive inter-kingdom interaction between bacteria and fungi for food foraging.

The data that have been generated for six healthy individuals sampled over a 2-month period provide a new vision of the link between the diet and the composition of the bacterial and fungal microbiomes. Altogether, our present work proposes an efficient workflow to study the human gut microbiome integrating robustly fungal, bacterial, and dietary data.

## Conclusion

The data that have been generated for six healthy individuals sampled over a 2-month period provide a new vision of the link between the diet and the composition of the bacterial and fungal microbiomes. Together, our present work proposes an efficient workflow to study the human gut microbiome integrating robustly fungal, bacterial, and dietary data. With this workflow, our findings demonstrated the interkingdom association between intestinal bacteria and fungi at taxonomic and functional levels and their correlation with diet.

### Supplementary Information

**Additional file 1: Data S1.** A total of 97 sequences in FASTA format were obtained from NCBI.

**Additional file 2: Data S2.** For creating the HMM of the SSU, 100 sequences were recovered and used through the same process as with the LSU.

**Additional file 3: Data S3.** The strains and their randomly attributed abundances.

**Additional file 4: Figure S1.** Workflow of generating simulated sequencing reads for mock communities. The colored dots represent the species in the in silico mock community. The genomes of the species were used directly as the input of InSilicoSeq for mimicking the shotgun sequencing. The ITS sequences of the species were replicated with their corresponding CN-MD before input to the InSilicoSeq for mimicking the ITS sequencing.

**Additional file 5: Figure S2.** Workflow diagram of the enrichment protocol, Created with BioRender.com.

**Additional file 6: Data S4.** This observation is not in agreement with a previous study that reported a high number of ITS copies, ranging from 14 to 1442.

**Additional file 7: Data S5.** The comparison between the two values did not show significant differences.

**Additional file 8: Data S6.** The resulting CN-MD ranged from 7 to 170, with an average of 60.

**Additional file 9: Data S7.** The resulting CN-MD ranged from 7 to 170, with an average of 60.

**Additional file 10: Figure S3.** PcoA plot of the expected mock community profiling and the recovered mock community profiling from shotgun sequencing and ITS sequencing.

**Additional file 11: Figure S4.** Total number of reads in each sample (a), and fungal reads in each sample with and without enrichment (b).

**Additional file 12: Figure S5.** Enrichment efficiency in bacteria.

**Additional file 13: Figure S6.** Genus level fungal microbiome community compositions grouped by different timepoints (a), and Genus level bacterial microbiome community compositions grouped by different timepoints (b).

**Additional file 14: Figure S7.** Bacterial taxonomic compositions are associated with habitual diet. Heatmap of all the detected significant correlations between bacterial taxonomic compositions and diet categories. The asterisk indicates that the correlation index for the corresponding species metadata pair is significant.

**Additional file 15: Figure S8.** Fungal functional compositions are associated with habitual diet. Heatmap of all the detected significant correlations between fungal functional compositions and diet categories. The asterisk indicates that the correlation index for the corresponding species metadata pair is significant.

**Additional file 16: Figure S9.** Bacterial functional compositions are associated with habitual diet. Heatmap of all the detected significant correlations between bacterial functional compositions and diet categories. The asterisk indicates that the correlation index for the corresponding species metadata pair is significant.

## Data Availability

Shotgun metagenomic sequencing raw data (short-read archives, SRA) are available via NCBI Project Number PRJNA925700, which includes the sequences from the 143 fecal samples described in the Methods section. The in-house scripts for performing bioinformatics analyses in this work can be found on GitHub at https://github.com/ManichanhLab/LongitudinalMycobiomeWithDiet.

## Abbreviations

ITS: Internal transcribed spacer
HMM: Hidden Markov model
CN: Copy number
MD: Mapping depth
SCMG: Single copy marker gene
SD: Standard deviation
Gbp: Giga base pair
FDR: False discovery rate
LSU: Large subunit
SSU: Small subunit
ICC: Intraclass correlation coefficient
sFFQ: Short food frequency questionnaire
RMT: Random matrix theory
