# Supplementary material for: Shotgun metagenomics reveals interkingdom association between intestinal bacteria and fungi involving competition for nutrients
Source: Microbiome. 2023 Dec 14;11:275. doi: 10.1186/s40168-023-01693-w (PMC10720197; doi:10.1186/s40168-023-01693-w)
Supplement: Supplementary file 5 — Additional file 4: Figure S1. Workflow of generating simulated sequencing reads for mock communities. The colored dots represent the species in the in silico mock community. The genomes of the species were used directly as the input of InSilicoSeq for mimicking the shotgun sequencing. The ITS sequences of the species were replicated with their corresponding CN-MD before input to the InSilicoSeq for mimicking the ITS sequencing. [file 40168_2023_1693_MOESM4_ESM.pdf]

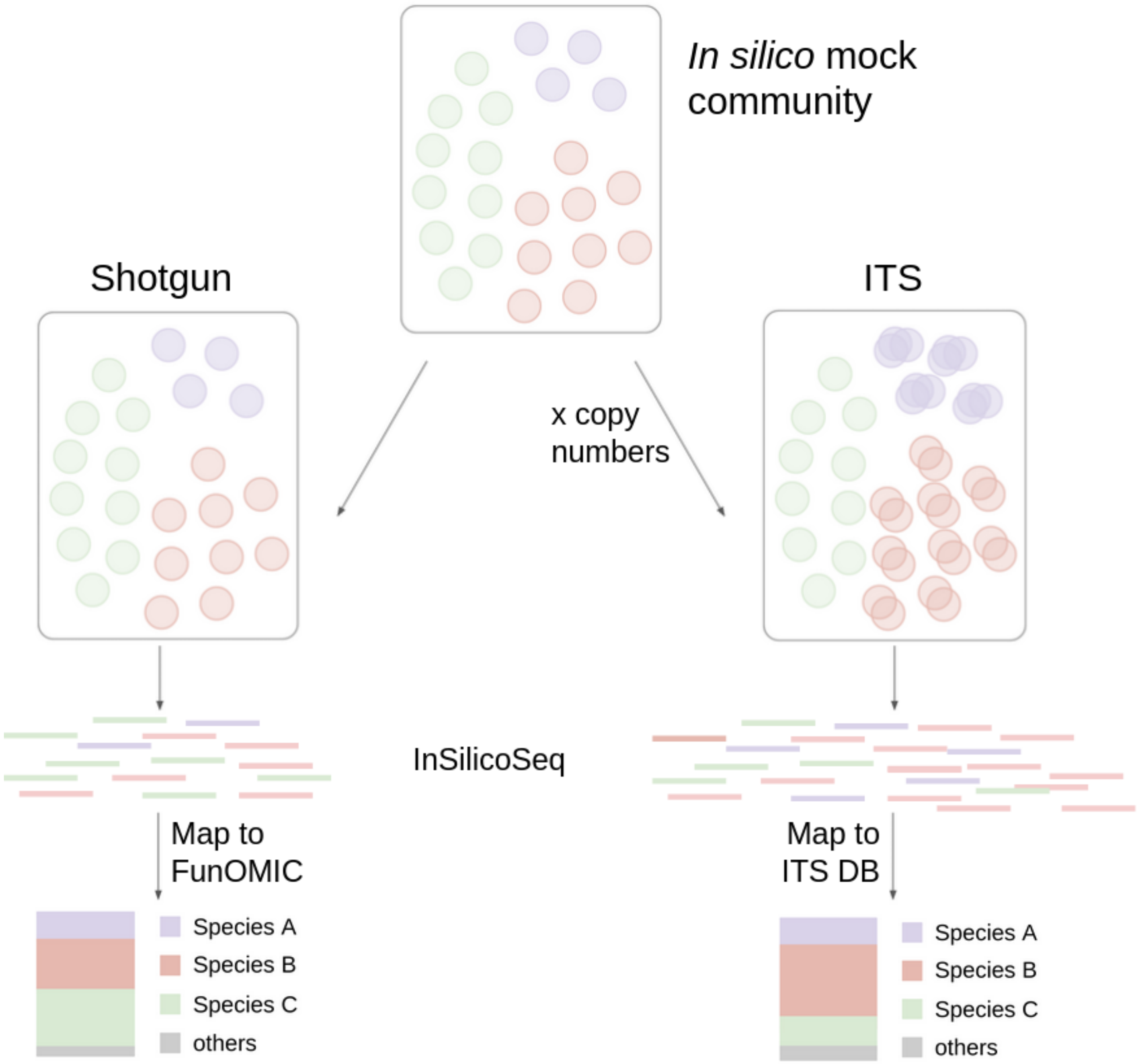

Supplementary Figure 5. Workflow of generating simulated sequencing reads for mock communities. The colored dots represent the species in the *in silico* mock community. The genomes of the species were used directly as the input of InSilicoSeq for mimicking the shotgun sequencing. The ITS sequences of the species were replicated with their corresponding CN-MD before input to the InSilicoSeq for mimicking the ITS sequencing.
