# Supplementary figures and images for: Shotgun metagenomics reveals interkingdom association between intestinal bacteria and fungi involving competition for nutrients
Source: Microbiome. 2023 Dec 14;11:275. doi: 10.1186/s40168-023-01693-w (PMC10720197; doi:10.1186/s40168-023-01693-w)

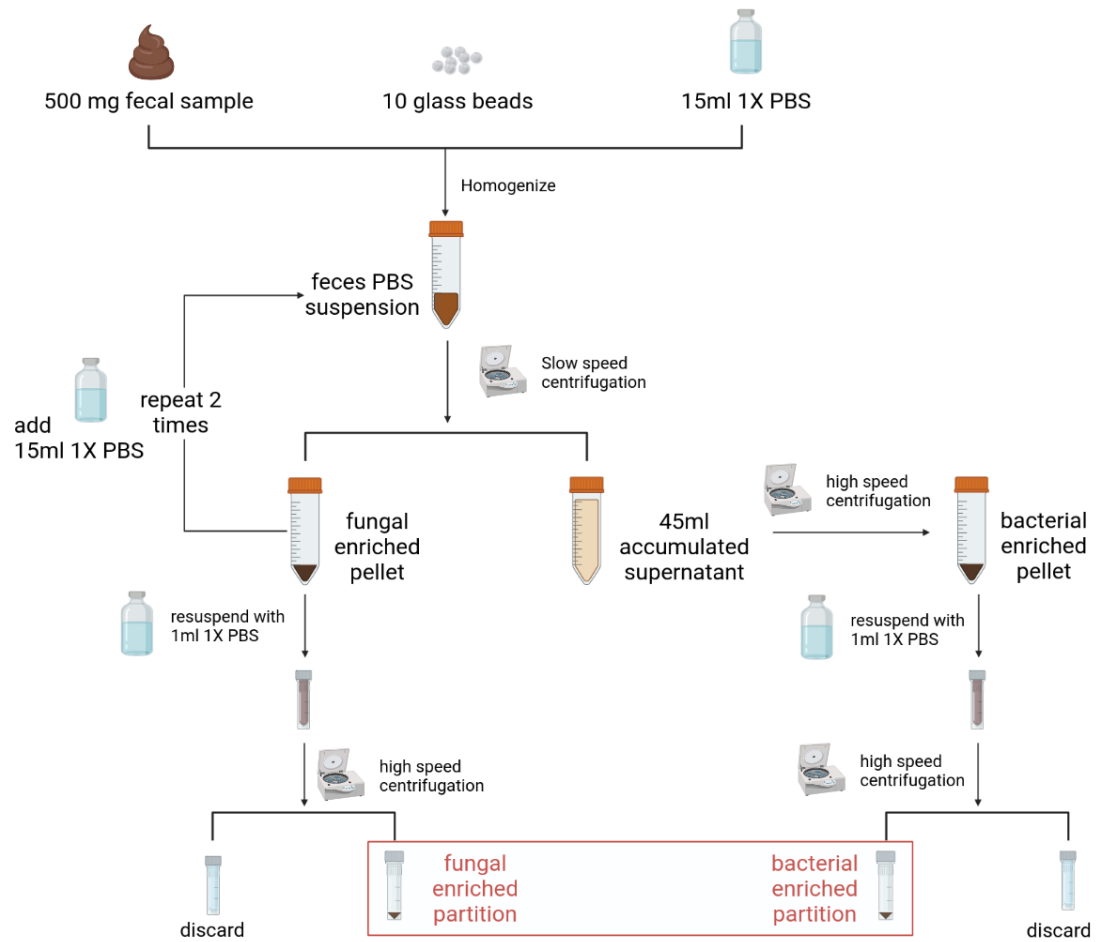

**Figure S2. Workflow diagram of the enrichment protocol, Created with BioRender.com.**

Supplement: Supplementary file 6 — Additional file 5: Figure S2. Workflow diagram of the enrichment protocol, Created with BioRender.com. [file 40168_2023_1693_MOESM5_ESM.pdf]
