# Supplementary material for: Shotgun metagenomics reveals interkingdom association between intestinal bacteria and fungi involving competition for nutrients
Source: Microbiome. 2023 Dec 14;11:275. doi: 10.1186/s40168-023-01693-w (PMC10720197; doi:10.1186/s40168-023-01693-w)
Supplement: Supplementary file 11 — Additional file 10: Figure S3. PcoA plot of the expected mock community profiling and the recovered mock community profiling from shotgun sequencing and ITS sequencing. [file 40168_2023_1693_MOESM10_ESM.pdf]

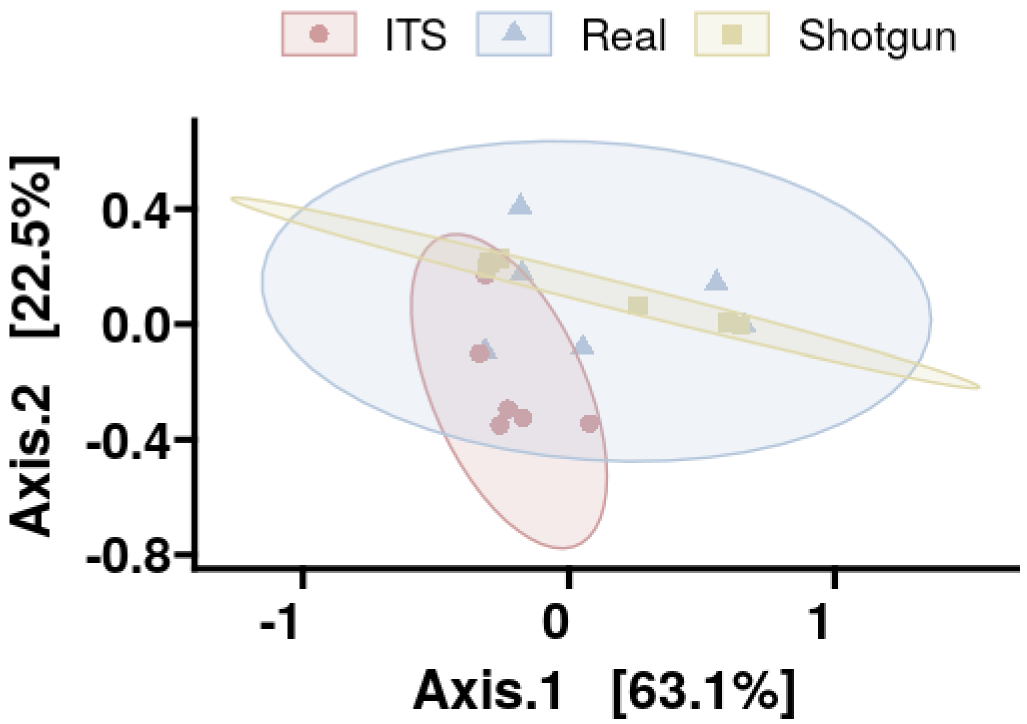

Figure S3. PcoA plot of the expected mock community profiling and the recovered mock community profiling from shotgun sequencing and ITS sequencing.
