# Supplementary material for: Shotgun metagenomics reveals interkingdom association between intestinal bacteria and fungi involving competition for nutrients
Source: Microbiome. 2023 Dec 14;11:275. doi: 10.1186/s40168-023-01693-w (PMC10720197; doi:10.1186/s40168-023-01693-w)
Supplement: Supplementary file 12 — Additional file 11: Figure S4. Total number of reads in each sample (a), and fungal reads in each sample with and without enrichment (b). [file 40168_2023_1693_MOESM11_ESM.pdf]

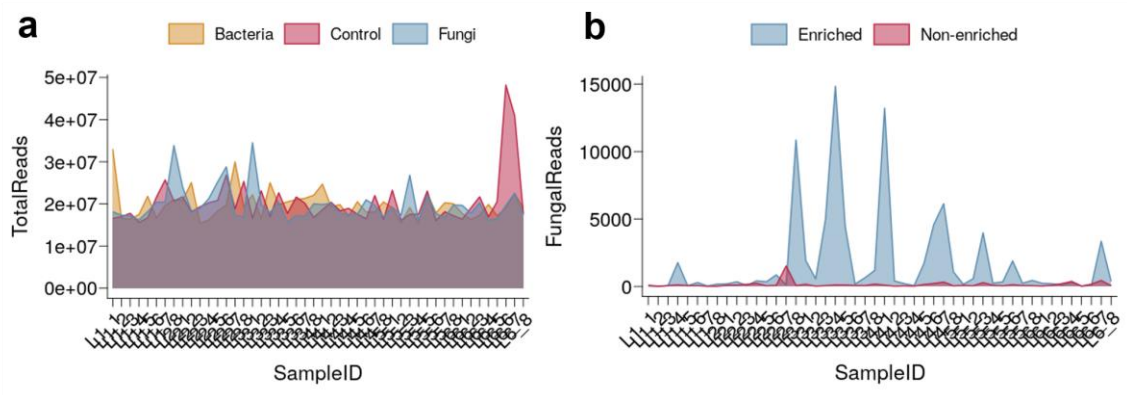


**Figure S4. Total number of reads in each sample (a), and fungal reads in each sample with and without enrichment (b).**
