# Supplementary material for: Shotgun metagenomics reveals interkingdom association between intestinal bacteria and fungi involving competition for nutrients
Source: Microbiome. 2023 Dec 14;11:275. doi: 10.1186/s40168-023-01693-w (PMC10720197; doi:10.1186/s40168-023-01693-w)
Supplement: Supplementary file 13 — Additional file 12: Figure S5. Enrichment efficiency in bacteria. [file 40168_2023_1693_MOESM12_ESM.pdf]

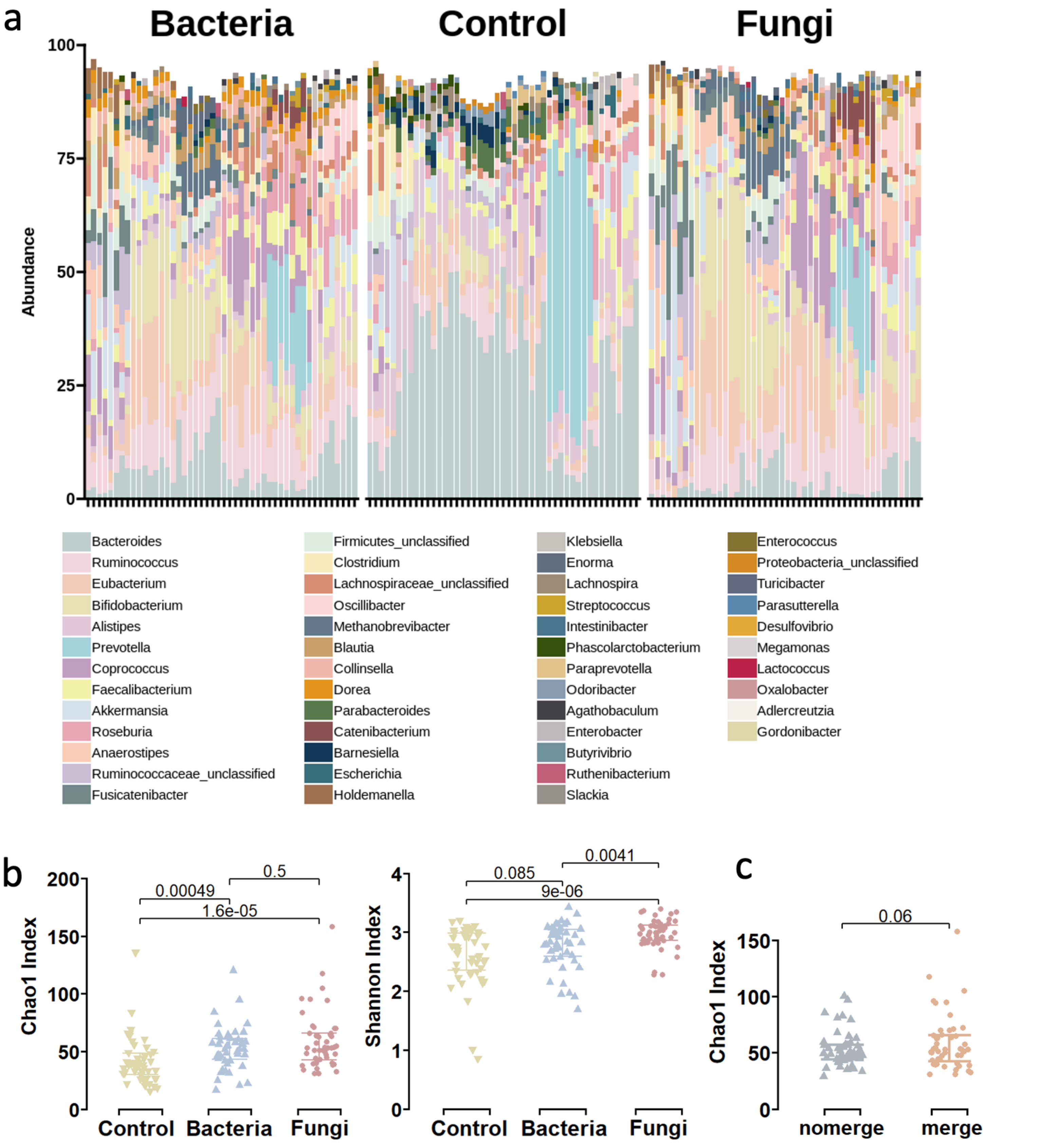

Supplementary Figure 1. Enrichment efficiency in bacteria. a, The genus-level taxa bar plot of the bacterial community compositions in bacterial, control, and fungal partitions. b, Boxplots of the species-level bacterial community alpha diversities (Shannon and Chao1 indices) in fungal, control, and bacterial partitions (n=143) ordered by their mean from smallest to largest (left to right). c, Boxplots of the species-level bacterial community alpha diversity (Chao1 index) before and after merging bacterial reads in the bacterial and fungal partitions (n=48) ordered by their mean from smallest to largest (left to right).
