# Supplementary material for: Shotgun metagenomics reveals interkingdom association between intestinal bacteria and fungi involving competition for nutrients
Source: Microbiome. 2023 Dec 14;11:275. doi: 10.1186/s40168-023-01693-w (PMC10720197; doi:10.1186/s40168-023-01693-w)
Supplement: Supplementary file 14 — Additional file 13: Figure S6. Genus level fungal microbiome community compositions grouped by different timepoints (a), and Genus level bacterial microbiome community compositions grouped by different timepoints (b). [file 40168_2023_1693_MOESM13_ESM.pdf]

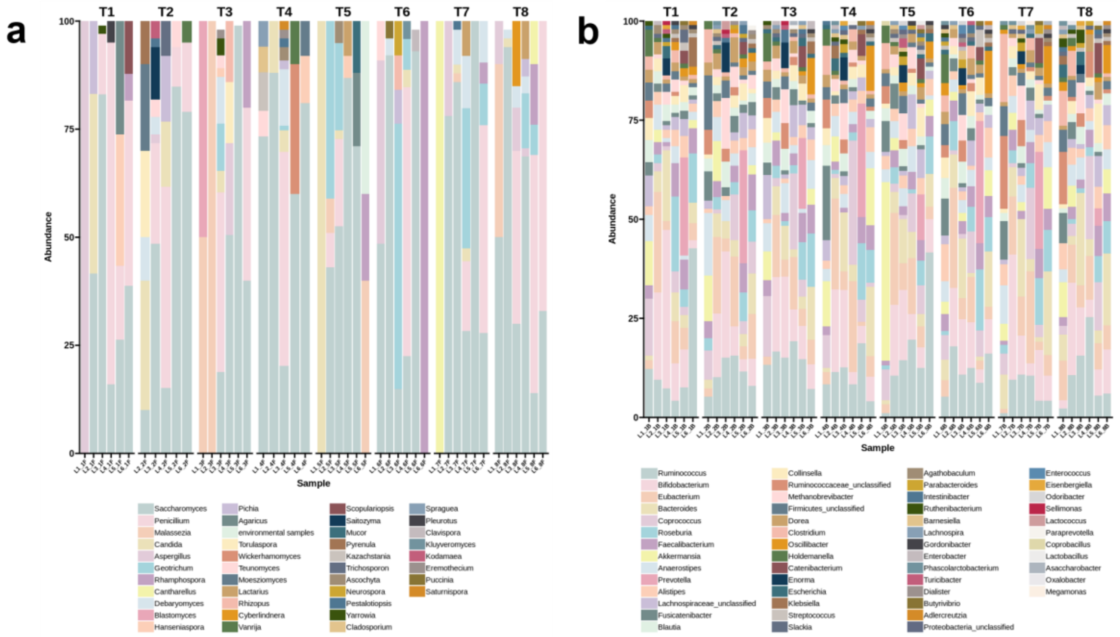

Figure S6. Genus level fungal microbiome community compositions grouped by different timepoints (a), and Genus level bacterial microbiome community compositions grouped by different timepoints (b).
