# Supplementary material for: Shotgun metagenomics reveals interkingdom association between intestinal bacteria and fungi involving competition for nutrients
Source: Microbiome. 2023 Dec 14;11:275. doi: 10.1186/s40168-023-01693-w (PMC10720197; doi:10.1186/s40168-023-01693-w)
Supplement: Supplementary file 15 — Additional file 14: Figure S7. Bacterial taxonomic compositions are associated with habitual diet. Heatmap of all the detected significant correlations between bacterial taxonomic compositions and diet categories. The asterisk indicates that the correlation index for the corresponding species metadata pair is significant. [file 40168_2023_1693_MOESM14_ESM.pdf]

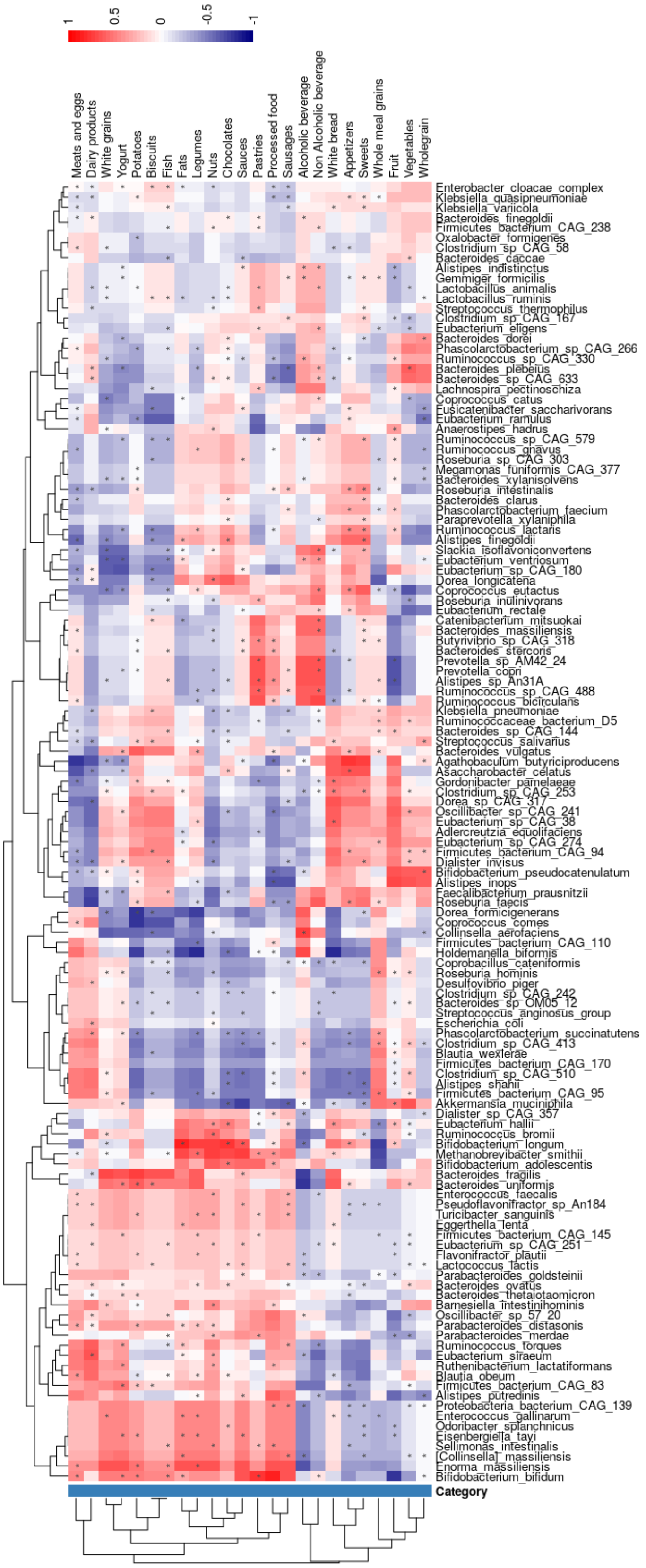

Supplementary Figure 7. Bacterial taxonomic compositions are associated with habitual diet. Heatmap of all the detected significant correlations between bacterial taxonomic compositions and diet categories. The asterisk indicates that the correlation index for the corresponding species metadata pair is significant.
