# Supplementary material for: Shotgun metagenomics reveals interkingdom association between intestinal bacteria and fungi involving competition for nutrients
Source: Microbiome. 2023 Dec 14;11:275. doi: 10.1186/s40168-023-01693-w (PMC10720197; doi:10.1186/s40168-023-01693-w)
Supplement: Supplementary file 16 — Additional file 15: Figure S8. Fungal functional compositions are associated with habitual diet. Heatmap of all the detected significant correlations between fungal functional compositions and diet categories. The asterisk indicates that the correlation index for the corresponding species metadata pair is significant. [file 40168_2023_1693_MOESM15_ESM.pdf]

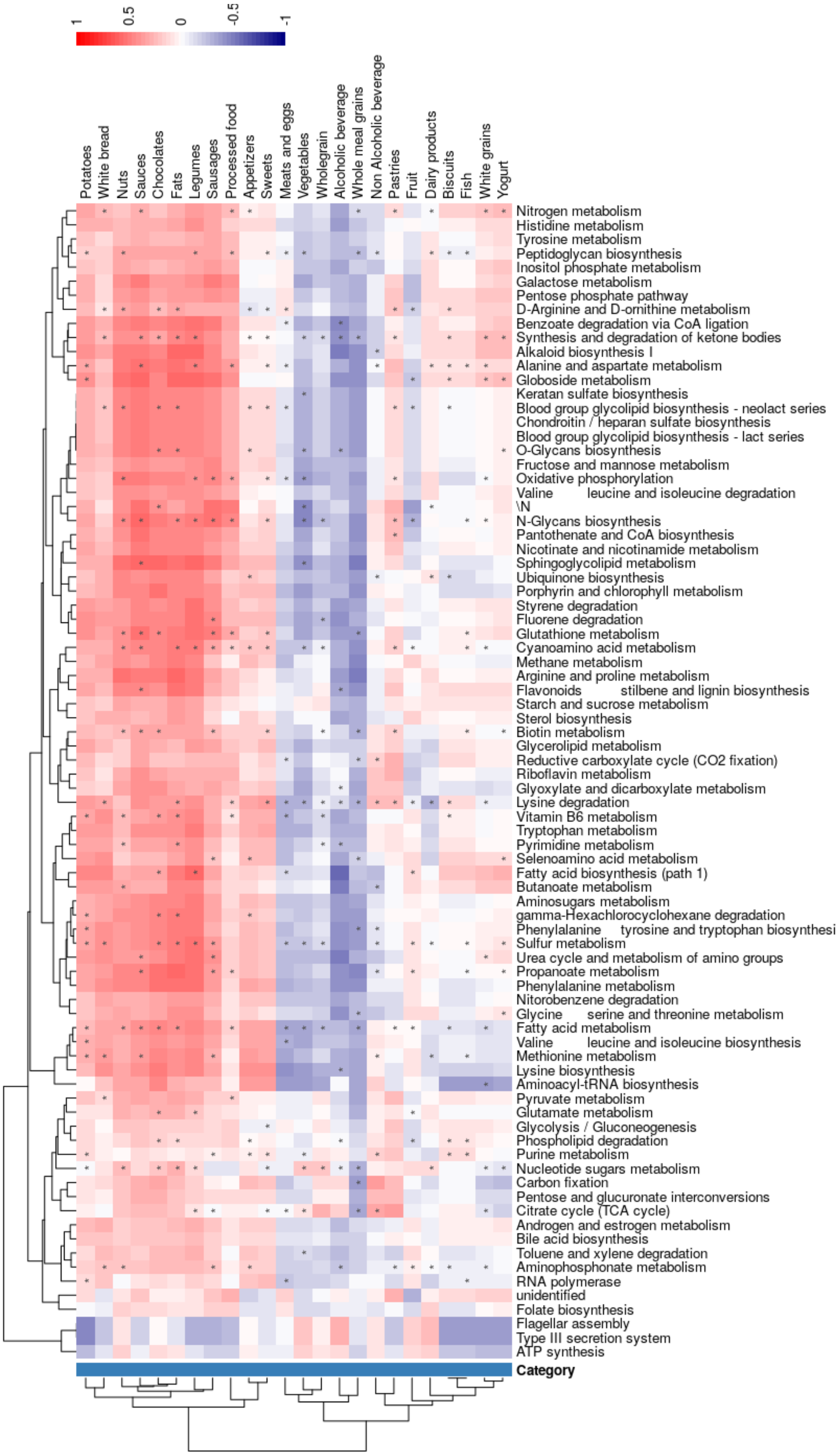

Supplementary Figure 8. Fungal functional compositions are associated with habitual diet. Heatmap of all the detected significant correlations between fungal functional compositions and diet categories. The asterisk indicates that the correlation index for the corresponding species metadata pair is significant.
